# Validating CircaCP: a generic sleep–wake cycle detection algorithm for unlabelled actigraphy data

**DOI:** 10.1098/rsos.231468

**Published:** 2024-05-29

**Authors:** Shanshan Chen, Xinxin Sun

**Affiliations:** ^1^ Department of Biostatistics, School of Population Health, Virginia Commonwealth University, Richmond, VA, USA; ^2^ Department of Biostatistics, School of Population Health, Virginia Commonwealth University, Richmond, VA, USA

**Keywords:** actigraphy, parametric change point detection, unsupervised approach, external validation

## Abstract

Sleep–wake (SW) cycle detection is a key step for extracting temporal sleep metrics from actigraphy. Various supervised learning algorithms have been developed, yet their generalizability from sensor to sensor or study to study is questionable. In this paper, we detail and validate an unsupervised algorithm—CircaCP—for detecting SW cycles from actigraphy. It first uses a robust cosinor model to estimate circadian rhythm, then searches for a single change point (CP) within each circadian cycle. Using CircaCP, we estimated sleep/wake onset times (S/WOTs) from 2125 individuals’ data in the MESA sleep study and compared the estimated S/WOTs against self-reported S/WOT event markers, using Bland–Altman analysis as well as variance component analysis. On average, SOTs estimated by CircaCP were 3.6 min behind those reported by event markers, and WOTs by CircaCP were less than 1 min behind those reported by markers. These differences accounted for less than 0.2% variability in S/WOTs, considering other sources of between-subject variations. Rooted in first principles of human circadian rhythms, our algorithm transferred seamlessly from children’s hip-worn ActiGraph data to ageing adults’ wrist-worn Actiwatch data. The generalizability of our algorithm suggests that it can be widely applied to actigraphy collected by other sensors and studies.

## 1. Introduction

Accurate sleep–wake (SW) cycle detection is essential for extracting temporal sleep metrics from actigraphy data [1]. Actigraphy records one’s activity levels at 30 s or 60 s intervals continuously for a prolonged time (e.g. a few days to several weeks). Using such records, researchers can estimate circadian cycles and SW cycles, and extrapolate sleep metrics such as sleep/wake onset times (S/WOTs), sleep durations and the within-subject variability of these sleep metrics.

Numerous algorithms for estimating SW cycles from actigraphy data have been developed and such research attempts date back to the 1980s [2,3]. Mullaney *et al.* were the first group to devise such algorithms and then Webster *et al.* proposed a scoring algorithm [3]. Other researchers extended Webster’s scoring method and devised their own scoring algorithm customized to their data [4–6]. These scoring algorithms have been widely used to identify SW states due to their accessibility and ease of use. In recent years, machine-learning techniques have also been applied in S/WOT detection. Most of these techniques are binary classification algorithms such as logistic regression [7–9], linear discriminant classifiers [10], support vector machines [11] and random forests [12,13].

**Figure 1.**
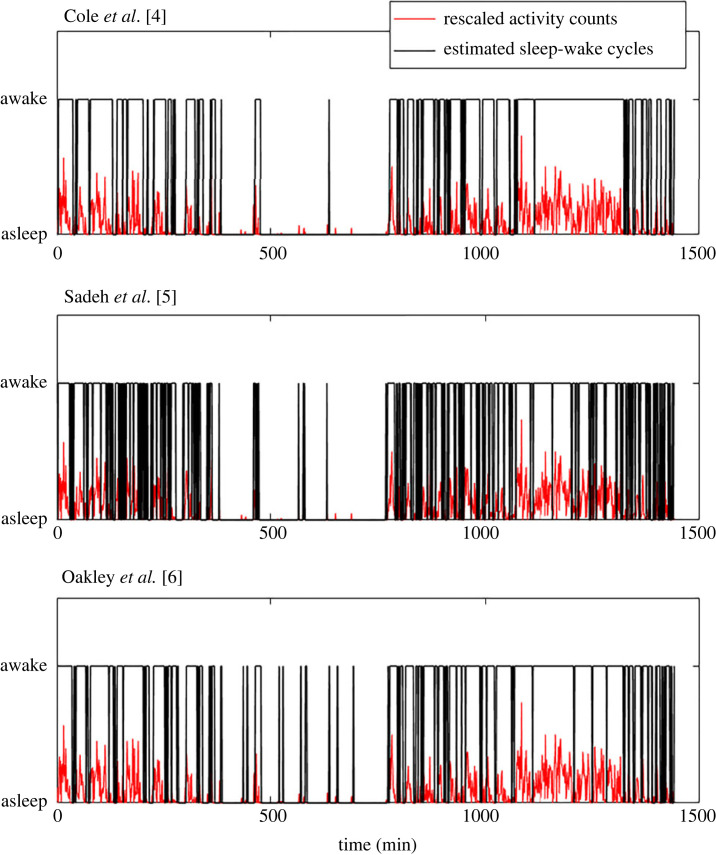
Sleep–wake states detected by three popular algorithms for one subject’s actigraphy data from the MESA sleep study.

These algorithms have several disadvantages. Firstly, algorithms based on generalized linear models or supervised machine-learning algorithms require ground-truth labels to train models for SW cycle detection. Labels for training models can be expensive to collect (e.g. from polysomnography (PSG) studies), contain inaccuracies (e.g. proxy reports), or be inconvenient to collect (e.g. researchers may elect not to collect labels in order to maximize the compliance rate). Secondly, since the debut of actigraphy sensors in the 1980s, activity levels have been measured in relative scales without a consistent unit. Thus, actigraphy data collected by different wearable activity trackers for the same types of activity have different ranges and distributions. As the parameters of these algorithms are fine-tuned to maximize the detection accuracy for a particular dataset, their generalizability to other datasets is questionable. Thirdly, these algorithms use fixed-length time windows to extract features, which cannot adequately capture the structural breaks in data distributions that may happen on various time scales [14]. Moreover, these algorithms assumed that the distributions of diurnal and nocturnal actigraphy are identical. As a result, the SW cycles estimated by these algorithms tend to have multiple misidentified sleep/wake states, resulting in multiple fragments of sleep/awake states. These estimated SW cycles may be further smoothed by an ad hoc temporal filter (e.g. a median filter) [15] or heuristic rules [3]. Although such filters may consolidate some of the fragments and improve detection accuracy [11], they can also further reduce the temporal resolution of the detection. Figure 1 illustrates this common issue, based on the application of three of these scoring algorithms to the actigraphy data of one subject.

Recognizing these issues, some researchers have devised algorithms focusing on general SW cycle detection. Van Hees *et al.* described an accelerometer-based estimation method without sleep labels; instead, heuristic cutoffs were used to estimate various postures and corresponding SW states [16]. Cakmak *et al.* fused wrist-worn sensor data collected at higher sampling rates, applied a binary segmentation algorithm to identify the change points (CP), and integrated three types of segmented time series by training generalized linear models [17]. These algorithms were designed in particular for wearable sensor data stored at higher temporal resolutions (e.g. 10 Hz), and cannot be readily applied to actigraphy data that are aggregated at 30 s or 1 min intervals. Hidden Markov models (HMMs) are an alternative to the supervised machine-learning models for segmenting the actigraphy sequences [18]. As a probabilistic model, training HMMs does not require as many ground-truth labels as required by the supervised alternatives. Instead, HMMs require *a priori* knowledge about the number of hidden states, initial parameters and data distribution assumption. False assumptions of HMMs can lead to sub-optimal estimation results. For example, Huang *et al.* [18] developed a three-state HMM with Gaussian assumption. To satisfy the Gaussian assumption, Huang *et al.* aggregated the minute-by-minute actigraphy data to 5 min epochs and used square-root transformation on the 5 min data and accepted the normality assumption (*p* = 0.109). Such transformation may not apply to actigraphy data at a higher resolution (e.g. 30 s or 1 min). Moreover, HMMs often require longer sequences for good estimation, which can be a problem when the sequences are shorter than 4 days.

So far, the existing SW detection algorithms have rarely been validated on a large, external population cohort. Moreover, most validation studies focused on validating against in-laboratory, one-night PSG data, omitting validation of days’ worth of actigraphy data collected in the free-living environment [11,19–22]. Samson *et al.* [23] were some of the few researchers who validated the Cole–Kripke algorithm [4] from actigraphy in the free-living environment on 33 subjects; however, they did not validate S/WOTs. This research gap prompts the question of how accurate SW cycle detection can be given unlabelled actigraphy data in the free-living environment.

Chen *et al.* [24] proposed a generic, unsupervised algorithm to detect SW cycles and estimate S/WOTs based on common characteristics in human circadian rhythms. The algorithm leverages the prior information obtained from a nonlinear parametric model, and detects the S/WOTs with higher precision using a parametric CP detection algorithm. This algorithm was tested on 112 children’s data collected from hip-worn ActiGraph sensors without ground-truth labels, and its generalizability has not yet been tested. In this paper, we detail the mathematical foundation of Chen’s algorithm [24], improve its computation time and robustness, and validate the algorithm on a large actigraphy dataset collected by wrist-worn Actiwatch sensors used in the Multi-Ethnic Study of Atherosclerosis (MESA) sleep study [25,26]. This dataset contains week-long actigraphy data from over 2000 adults of diverse ages, ethnicities and work schedules, and are accompanied with self-reported S/WOTs recorded as event markers, making them perfect for external validation. We name this improved and validated SW cycle detection algorithm CircaCP—a circadian rhythm guided CP detection method. An open-source Matlab implementation of CircaCP can be found at https://github.com/ShanshanChen-Biostat/CircaCP.

## 2. Methods

### 2.1. Data collection

A total of 2237 subjects aged from 45 to 84 were enrolled in the MESA sleep study between 2010 and 2012. During the study, all 2237 subjects were instructed to place an Actiwatch (Philips Respironics, Inc.) on the non-dominant wrist, and wear the same sensor for at least 5 days in the habitual living environment. Subjects were also asked to press the small button located on the right side of the Actiwatch every time they went to sleep or woke up, creating event markers. After the study days, the devices were returned to the study staff, and the data from these devices were downloaded by Respironics Actiware 5 Software, and aggregated as activity counts in 30 s intervals using the Actiware-Sleep software. The quality of these data was scored by the Sleep Reading Center at the Brigham and Women’s Hospital.

### 2.2. Measures

A total of 2159 subjects in the MESA sleep study had actigraphy data, of whom 2125 had a minimum of three days of data that were no less than 50% reliable. These 2125 subjects entered our actigraphy analysis. The quality of these actigraphy data was rated from 2 to 7 (2 being the poorest and 7 being the best, named as G2 (poor), G3 (fair), G4 (good), G5 (very good), G6 (excellent), G7 (outstanding) in the rest of this paper) according to consistency with PSG data, event markers, sleep diary and light levels (more details can be found in the MESA Exam 5 Sleep Data Documentation Guide from sleepdata.org/datasets/mesa). Note that quality grades do not necessarily reflect the quality of the actigraphy data alone, but also depend on the completeness and quality of sleep diaries. We used a one-dimensional time series of the actigraphy data—the vector magnitude of activity counts—for SW cycle detection, and compared the detection results with the event marker data to validate the SW cycles detected by our algorithm.

### 2.3. Screening of actigraphy data

To reliably estimate circadian rhythms, we included subjects who had worn the sensor continuously for at least four days. To do so, we first excluded subjects with actigraphy of less than 5760 min. Then among the remaining subjects, we detected the continuous wearing periods, defined as having no consecutive zeros more than 120 min. We included subjects with a longest continuous wearing period of at least 5760 min. Lastly, we aggregated the included 30 s interval actigraphy into 60 s interval actigraphy.

### 2.4. Sleep–wake cycle detection algorithm

This section details Chen’s algorithm for SW cycle detection. The algorithm consists of two steps: (i) segmenting the time series of actigraphy data into circadian cycles using a nonlinear parametric model, each cycle consisting of a diurnal and a nocturnal period and (ii) identifying more accurate sleep-to-wake and wake-to-sleep CPs within each segment.

The time series of activity data demonstrate a strong pseudo-periodical pattern (i.e. circadian rhythm), which can thus be modelled by functions with periodical patterns, e.g. a cosine function. Halberg *et al.* [27] proposed a cosinor model to fit data with circadian patterns. It has three parameters, amplitude *amp* which is the peak of the rhythm, *mes* which indicates the midline estimating statistic of rhythm, and acrophase *ϕ* which is the time of the peak of the rhythm, to be estimated to identify the circadian rhythm. The cosinor model is shown in equation (2.1):

2.1 $$r(t)=mes+amp\times \cos\left(\frac{[t-\phi ]\times 2\pi }{T}\right),$$

where *t* = 1, 2, …, *n*, t∈N, is the time index for the actigraphy sequence. *T* is the period of one’s circadian rhythm, often set as a constant of 24 (hours), or in our case 1440 (minutes). Marler’s extension of this model, the sigmoidally transformed cosine model, can also be used to capture the circadian rhythm. Since the cosinor model takes significantly less computation time without sacrificing much accuracy, our study implemented the cosinor model to fit the nonlinear curve. We estimated the parameters of the nonlinear curve by minimizing the sum of squared residuals. The objective function we optimized is displayed in the formula below:

2.2 $$\hat{p}=\underset{p\in R^{3}}{\mathrm{argmin}}\;\|F(p,t)-Y\|,$$

where **p** is a three-parameter vector containing *mes*, *amp* and *ϕ* in equation (2.1). The initial value of **p** is set as (500, 550, 227). *F*(**p**, *t*) is the fitted curve and *Y* the raw activity counts with sequence index *t*, t∈N. After the nonlinear curve fitting, we dichotomize the fitted curve *F* by treating the lower 18% of its range as nocturnal periods and the upper 82% as diurnal periods. This cutoff is chosen based on the fact that in ageing population, the proportion of sleep is about 20%. This dichotomized curve gives an estimate of one’s circadian cycles whose transition edges are rough estimates of S/WOTs.

The fixed period of the cosinor model imposes consistent S/WOT over the days and hence cannot capture day-to-day variation in S/WOT for each individual. Therefore, the precise sleep/wake times must be determined with a more refined searching algorithm. Next, we locate more precise S/WOTs for a particular circadian cycle identified by the cosinor model. If a subject’s actigraphy sequence has clear circadian patterns, we assume the subject gets up at least once between two consecutive SOTs, or goes to sleep at least once between two consecutive WOTs, during a day. As a subject’s activity pattern shifts most drastically from night to day or day to night, we can assume that S/WOTs are the most significant structural break points before and after which the data distributions were drastically different. Such structural CPs can be detected using a parametric CP detection method.

**Figure 2.**
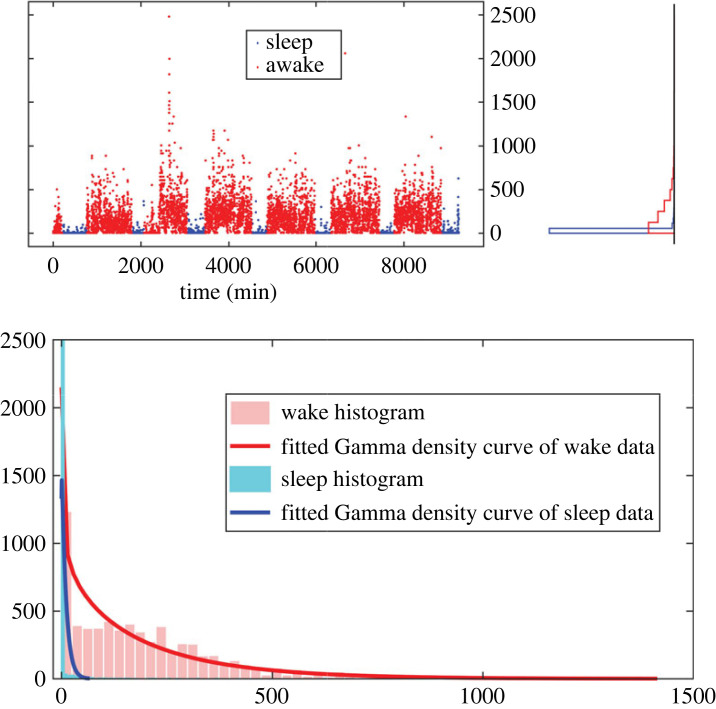
Histograms of one subject’s actigraphy data by sleep period and awake periods. Actigraphy data within each period were fitted by Gamma distributions.

Although count data are traditionally modelled by Poisson distributions, activity ‘counts’ are not natural counts that are generated from a counting process, but intensity measures of activity based on various algorithms. Distributions of such intensity data (e.g. time series of seismic moments and precipitation intensity) usually show long-tail, zero-inflated patterns, and have been modelled by Gamma distributions [28,29]. Thus, we fit Gamma distributions to MESA actigraphy data, as shown in figure 2. Activity distributions during waking and sleep are distinctively different: the activity counts during sleep have a higher density at 0 compared to those during waking periods.

Thus, we describe the time series of actigraphy as a pseudo-cyclostationary random process *Y*, which collects a sequence of independent and identically distributed random variables, *Y* = [*y*_1_, *y*_2_, …, *y*_*k*_, …, *y*_*n*_], that follow Gamma distributions, i.e.

2.3 $$y_{k}∼\mathrm{Gamma}(\theta_{k},\xi ),$$

where *θ*_*k*_ is the scale parameter for the random variable at time *k*, *y*_*k*_ and *ξ* is the shape parameter shared by the random variables [*y*_1_, *y*_2_, …, *y*_*k*_, …, *y*_*n*_]. A structural CP in such a time series means the scale parameter of the sequence before and after this point is significantly different. To test if such a structural CP exists in a segment of time-series data, we perform a test based on likelihood ratios. The hypothesis can be expressed by the following mathematical notation:

2.4 $$H_{0}\;:\;\theta_{1}=\theta_{2}=\cdots =\theta_{k}=\cdots =\theta_{n}=\theta_{0}$$

and

2.5 $$H_{1}\;:\;\theta_{0}=\theta_{1}=\theta_{2}=\cdots =\theta_{k}\neq \theta_{k+1}=\cdots =\theta_{n},$$

where *k* is the location of the CP, *n* the length of the data sequence, and *δ* a real number. Under *H*_0_, the likelihood function is

2.6 $$L_{0}=\frac{\prod_{i=1}^{n}y_{i}^{\xi -1}}{\Gamma^{n}(\xi )}\frac{1}{\theta_{0}^{n\xi }}\exp\frac{-\sum_{i=1}^{n}y_{i}}{\theta_{0}},$$

whereas under *H*_1_, the likelihood function is

2.7 $$L_{1}(\theta_{1},\theta_{2})=\frac{\prod_{i=1}^{n}y_{i}^{\xi -1}}{\Gamma^{n}(\xi )}\frac{1}{\theta_{1}^{k\xi }}\frac{1}{\theta_{2}^{(n-k)\xi }}\cdot \exp\frac{-\sum_{i=1}^{k}y_{i}}{\theta_{1}}\cdot \exp\frac{-\sum_{i=k+1}^{n}y_{i}}{\theta_{2}},$$

where *j* = 1, 2, …, *k*, …, *n* − 1, and represents all the possible locations of a CP *k*. Thus, to estimate the location of the CP *k* in a time series, our goal is to find the maximum-likelihood ratio *L*_1_/*L*_0_, or the minimum of the Bayesian information criteria (BIC). Chen *et al.* [30] proposed improved criteria, called modified information criteria (MIC), which is particularly suited for assessing model complexity in CP models. Thus, here we adapted the MIC term with a scaling factor *λ*, where *λ* controls the penalty of the CP being detected near the beginning or near the end of a sequence:

2.8 $$\begin{array}{rl} \mathrm{MIC}(k) & =-2\log(L_{1}(\hat{\theta_{1}},\hat{\theta_{2}}))+2\log n\\ & =2k\xi \log\sum_{i=1}^{k}y_{i}+2(n-k)\xi \log\sum_{i=k+1}^{n}y_{i}-2k\log k\xi -2(n-k)\log(n-k)\xi \\ & \;-2(\xi -1)\sum_{i=1}^{n}\log y_{i}+2\log n+\lambda \times {\left(\frac{2k}{n}-1\right)}^{2}\log n. \end{array}$$

Since the third and fifth terms in equation (2.8) do not contain location parameter *k*, we dropped the fifth and sixth terms from the model and simplified the fourth term. Therefore, equation (2.8) can be reduced to

2.9 k^=argmink∈N⁡ [2kξlog⁡∑i=1kyi+2(n−k)ξlog⁡∑i=k+1nyi∑i=k+1n−2kξlog⁡(nξ)−2(n−k)ξlog⁡((n−k)ξ)+λ×(2kn−1)2log⁡n],

$\text{where }\hat{k}$ is the estimated location of the CP in sequence *Y*. *λ* is empirically determined as 50. The whole algorithm for SW cycle detection is detailed in algorithm 1. A Matlab implementation of CircaCP can be found at https://github.com/ShanshanChen-Biostat/CircaCP.

**Table:** 

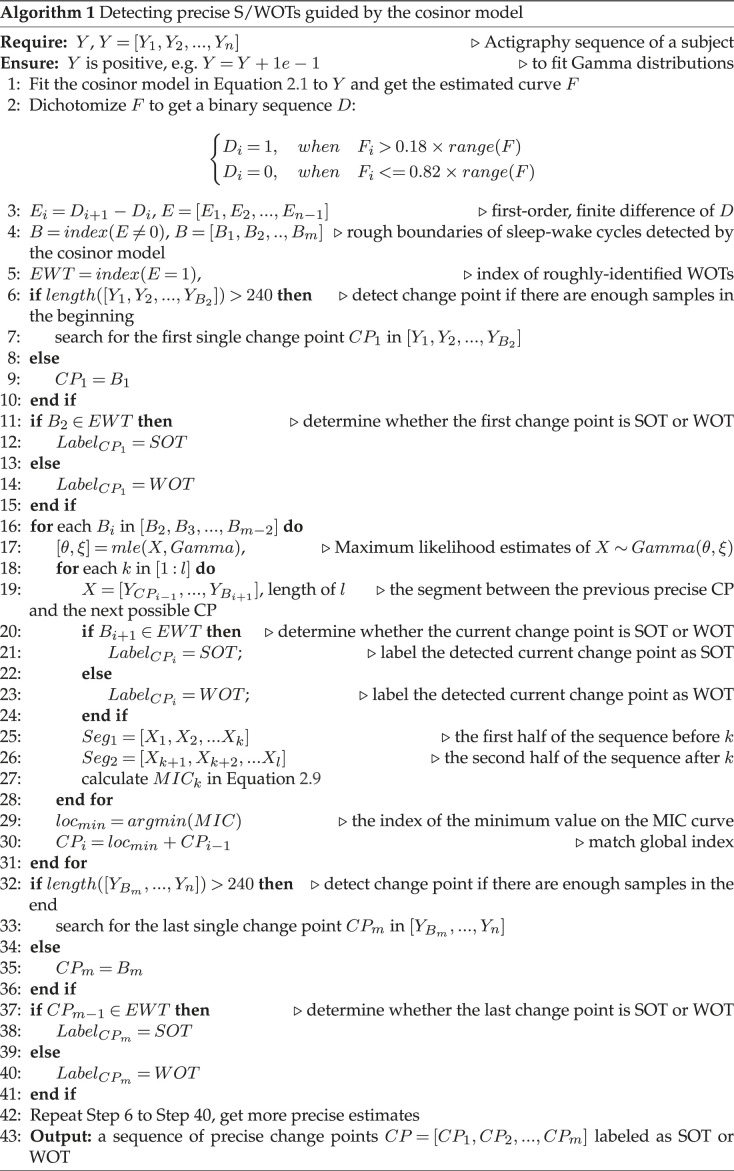

### 2.5. Error detection

To identify detection errors without ground-truth labels, we adopted a metric for evaluating clustering performance—the Calinski–Harabasz (CH) criterion [31]. Also known as the variance ratio criterion, this metric measures the ratio of total between-cluster sum of squares (SSB) to total within-cluster sum of squares (SSW):

2.10 $$\mathrm{CH}=\frac{\mathrm{SSB}}{\sum_{i=1}^{k}{\mathrm{SSW}}_{k}}\times \frac{n-k}{k-1},$$

where *n* is the total number of samples in an actigraphy sequence and *k* is the number of clusters, in our case, *k* = 2 (i.e. sleep and awake periods). We evaluated both the CH for SW cycles estimated by the cosinor model (CH_cos_) and by our proposed algorithm (CH_pro_). Detection results with CH_pro_ − CH_cos_ < 100 were identified as containing detection errors and removed from the subsequent analysis.

### 2.6. Validation procedure

Self-reported event markers contain inherent errors. For example, the subject may forget to click the button before falling asleep or after waking up, or click the button multiple times for a true S/WOT, or even at a random time of day which was not bed time or wake-up time. Thus, for validation, we paired up the S/WOTs detected by the CircaCP algorithm with the most likely corresponding event markers. First, sequence alignment methods such as the Needleman–Wunsch algorithm [32] and dynamic time warping [33] were applied to match event markers and the estimated S/WOTs. However, these existing alignment methods did not work well because long gaps between event markers and S/WOTs are quite common when the subject forgets to record S/WOTs. On the other hand, the correct event markers can always be detected close to true S/WOTs. Hence, we searched for event markers within the proximity of the estimated S/WOTs directly. Here, we defined a marker as valid if it fell within 180 min of the estimated S/WOT. If multiple markers matched for a certain S/WOT, we kept only the latest marker for SOT and the earliest marker for WOT. Once the event markers and the estimated S/WOTs were matched, we converted the index-based S/WOTs and event markers to minute-based S/WOTs, defined as the time elapsed since midnight, for quantitative analysis. SOTs occurring after midnight had 1440 min added to the original elapsed time.

To assess the variability in S/WOTs from the two sources (i.e. event markers and CircaCP estimation) and from actigraphy of different quality, we conducted variance component analyses to decompose the total variance in the data into the percentage contributions of various random effects (i.e. algorithm, subject and within-subject, day-to-day variability). If the majority of variance comes from within-subject variability (due to the natural day-to-day variability) and between-subject variability (due to subject characteristics, e.g. demographics, work schedules or disease types), as opposed to the algorithm type (i.e. either event markers or our algorithm), it means our algorithm is equivalent to the event markers in a population study. We also assessed the effects of these factors on the outcome—SOT or WOT—using linear mixed-effects models. Specifically, actigraphy quality group, algorithm, work schedule, age, gender and ethnicity were modelled as fixed effects. The reference levels were the highest actigraphy quality group (i.e. G7), event markers, being white, female and youngest, and having a day job. Day-to-day variability was modelled as random effects nested within-subjects. Lastly, we assessed the agreement between the two measurement methods using Bland–Altman analysis and median absolute errors.

## 3. Results

Out of the 2125 subjects’ actigraphy data, 1957 subjects’ actigraphy passed the screening criteria (i.e. having a continuous wearing time of at least 4 days) and were used for SW detection (table 1). Our screening criteria kept more than 90% of subjects’ actigraphy of varying quality, with quality group G7 having the most subjects included. Among these 1957 subjects, 34 subjects with poor SW detection results (evaluated by the error detection procedure) were excluded. Of the remaining 1923 subjects, 1857 had at least one matched marker and algorithm-detected S/WOT pair and entered the validation analysis. Statistics of the matching between event markers and estimated S/WOTs from the 1923 subjects are presented table 2. In total, 34 subjects did not push event buttons at all. Besides these 34 subjects, another 32 subjects who had no event marker matching estimated S/WOTs by CircaCP were excluded from analysis. On average, WOTs matched (74%) slightly more than the SOTs (72%) with the event buttons. Matching also differed among different actigraphy quality groups, with the best actigraphy quality group (G7) having the most event marker matching (89% for SOTs and 86% for WOTs), and the worst actigraphy quality group having the least event markers matching (58% for SOTs and 62% for WOTs). The demographics of the 1857 subjects who entered further analysis are listed in table 1. The majority of these subjects were retired, aged between 66 and 71, white, and female. In the group with the lowest actigraphy quality (G2), subjects were older and mostly African-American, and had a higher percentage of shift jobs than other groups. The G2 group had the lowest percentage (88.9%) of actigraphy that entered the validation analysis, whereas in the G4 to G7 groups, almost all actigraphy sequences for SW detection entered the validation analysis.

**Table 1.** Summary of actigraphy data in MESA sleep study.

	actigraphy quality group					
	G2 (poor)	G3 (fair)	G4 (good)	G5 (very good)	G6 (excellent)	G7 (outstanding)
original no.	692	573	49	132	580	99
no. for SW detection	627	525	46	121	544	94
no. with poor SW results	15	13	0	2	3	1
no. with valid SW results	612	512	46	119	541	93
included for validation	*N* = 558	*N* = 503	*N* = 45	*N* = 119	*N* = 539	*N* = 93
age						
mean (s.d.)	71.2 (9.22)	69.7 (9.36)	68.2 (8.24)	69.6 (9.19)	68.5 (8.50)	67.7 (8.48)
median [min, max]	71.0 [55.0, 94.0]	69.0 [54.0, 94.0]	68.0 [55.0, 86.0]	68.0 [55.0, 92.0]	67.0 [55.0, 91.0]	66.0 [55.0, 88.0]
gender						
female	296 (53.0%)	292 (58.1%)	26 (57.8%)	53 (44.5%)	280 (51.9%)	49 (52.7%)
male	262 (47.0%)	211 (41.9%)	19 (42.2%)	66 (55.5%)	259 (48.1%)	44 (47.3%)
race						
White Caucasian	127 (22.8%)	177 (35.2%)	20 (44.4%)	49 (41.2%)	287 (53.2%)	62 (66.7%)
Chinese American	58 (10.4%)	50 (9.9%)	6 (13.3%)	16 (13.4%)	54 (10.0%)	6 (6.5%)
African American	237 (42.5%)	155 (30.8%)	6 (13.3%)	23 (19.3%)	94 (17.4%)	14 (15.1%)
Hispanic	136 (24.4%)	121 (24.1%)	13 (28.9%)	31 (26.1%)	104 (19.3%)	11 (11.8%)
work schedule						
day shift	122 (21.9%)	156 (31.0%)	18 (40.0%)	41 (34.5%)	188 (34.9%)	37 (39.8%)
afternoon shift	25 (4.5%)	7 (1.4%)	0 (0%)	3 (2.5%)	12 (2.2%)	0 (0%)
irregular shift	25 (4.5%)	36 (7.2%)	3 (6.7%)	4 (3.4%)	33 (6.1%)	12 (12.9%)
night shift	8 (1.4%)	6 (1.2%)	0 (0%)	1 (0.8%)	9 (1.7%)	1 (1.1%)
split shift	8 (1.4%)	6 (1.2%)	1 (2.2%)	0 (0%)	6 (1.1%)	0 (0%)
rotating shift	6 (1.1%)	6 (1.2%)	0 (0%)	0 (0%)	2 (0.4%)	2 (0.4%)
retired	364 (65.2%)	286 (56.9%)	23 (51.1%)	70 (58.8%)	289 (53.6%)	41 (44.1%)

**Table 2.** Event button markers matching results.

	actigraphy quality group						
	G2	G3	G4	G5	G6	G7	overall
percentage of SOT matching (%)	58	72	79	79	83	89	72
percentage of WOT matching (%)	62	76	77	79	82	86	74
no. of subjects who did not push buttons at all	28	3	1	0	2	0	34

**Figure 3.**
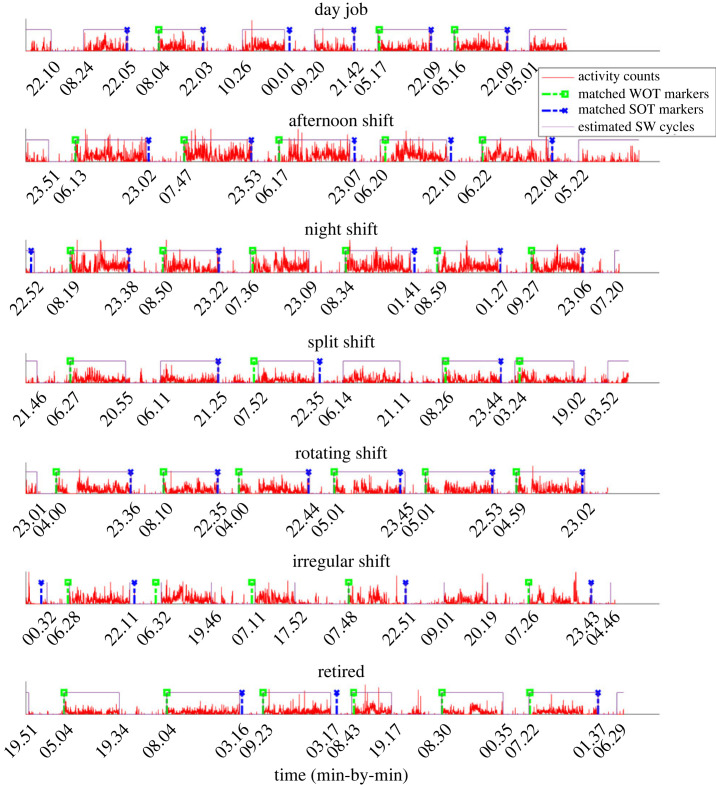
Visualization of SW detection results by CircaCP.

**Figure 4.**
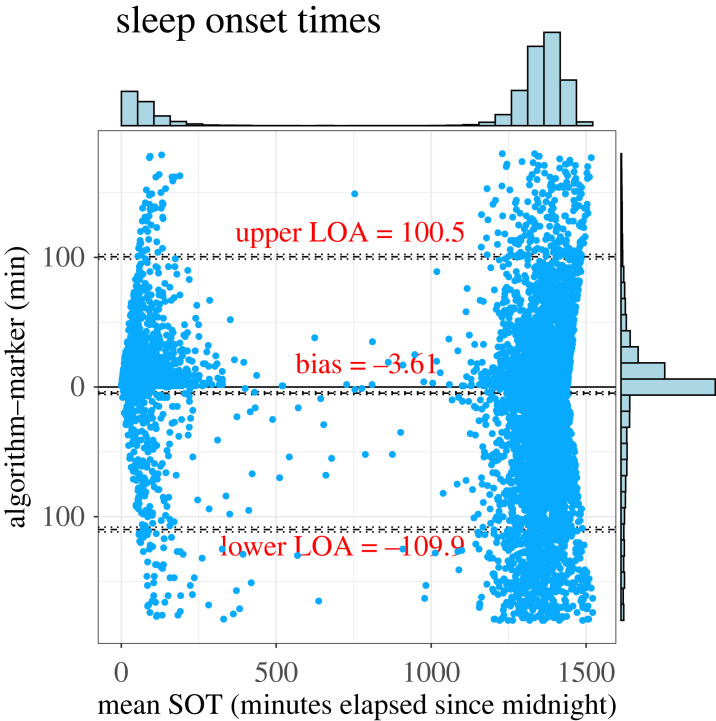
Bland–Altman plot for SOTs. The horizontal histogram shows the distribution of SOTs and the vertical histogram on the right is the error distribution. LOA indicates limit of agreement. *X*-axis is the mean of SOTs estimated by our algorithm and reported by event markers, serving as a measure of the true values.

**Figure 5.**
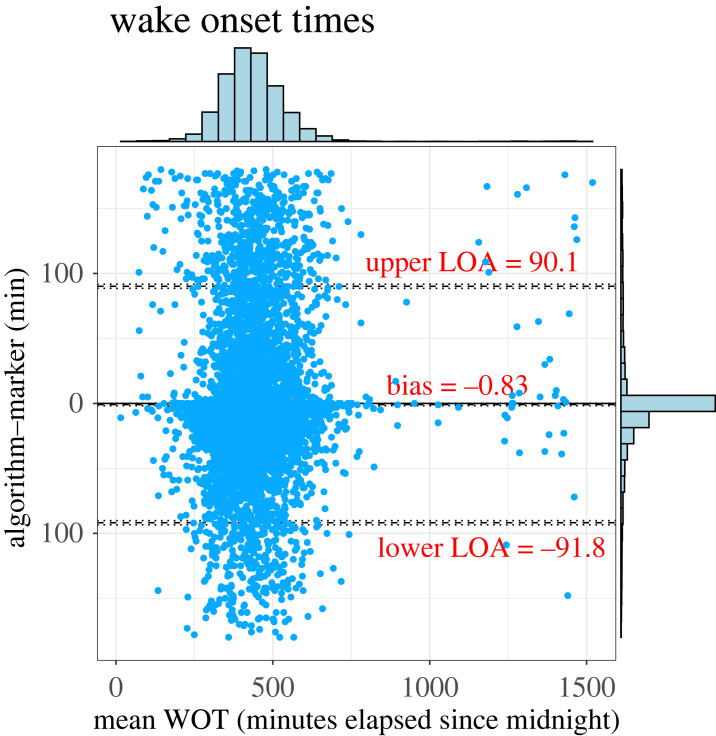
Bland–Altman plot for WOTs. The horizontal histogram shows the distribution of WOTs and the vertical histogram on the right is the error distribution. LOA indicates limit of agreement. *X*-axis is the mean of WOTs estimated by our algorithm and reported by event markers, serving as a measure of the true values.

The SW cycle detection results are visualized in figure 3. Despite the variety of work schedules, the CircaCP algorithm correctly identified SW cycles, where the transition edges are the estimated S/WOTs. These S/WOTs are very close to the S/WOTs reported by the event markers. Overall, SOTs and WOTs estimated by our algorithm have a median absolute error of 13 min and 7 min, respectively, against event markers (table 3). The SW detection accuracy also increased with actigraphy quality: the G7 group had the lowest mean absolute error (MAE; 5 min in SOT and 3 min in WOT) and the G2 group had the highest MAE (29 min in SOT and 17 min in WOT). Bland–Altman plots in figures 4 and 5 show the limits of agreement between the estimated S/WOTs and those recorded as the event markers. Although our marker matching procedure implied that the maximum difference between matched markers and estimated S/WOTs is ±180 min, the limits of agreement between the two were well under 180 min (about 100 min for SOT and 90 min for WOT). Our variance-component model further analyses the factors that impact the S/WOT outcomes (table 4). Overall, the factor of S/WOT estimation method contributes less than 0.2% to the total S/WOTs variance, suggesting that the CircaCP algorithm is equivalent to the event markers when analysing the impact of the estimation algorithm on the outcomes. The majority of the variation comes from the subject-to-subject variability and day-to-day variability.

**Table 3.** Median absolute errors (minutes) of S/WOT estimated by the proposed algorithm against event markers.

	actigraphy quality group						
	G2	G3	G4	G5	G6	G7	overall
SOT	29	16	10	10	7	5	13
WOT	17	8	6	8	4	3	7

**Table 4.** Results of variance component analysis. All numbers are presented as percentages.

	SOT	WOT
algorithm	0.154	0.002
subject	59.122	43.736
subject/day	33.675	47.599
residual	7.049	8.663

Results from linear mixed-effects models are shown in table 5. SOTs estimated by our proposed algorithm are about 6 min behind SOTs reported by markers in the highest-quality actigraphy group, and more behind (though non-significantly) in worse-quality actigraphy groups. WOTs detected by CircaCP are almost identical to those recorded by markers, with a non-significant difference of less than 1 min. On average, the subjects who are the youngest white females with a day job, and have the best actigraphy quality slept at 23.23 and woke up at 6.45. Having a non-regular work schedule significantly affects the S/WOT outcomes, and working night shifts delays WOTs the most among all types of schedule. Among the four race groups, African Americans had significantly delayed SOTs in comparison to white Caucasians. Since the quality grades do not entirely reflect raw actigraphy quality, this factor (i.e. the quality groups) did not impact the S/WOT outcomes significantly.

**Table 5.** Results from linear mixed-effects models. Significance level set at *p* < 0.05 shown in italics.^a^

	SOT			WOT		
predictors	estimates	CI	*p*-value	estimates	CI	*p*-value
(intercept)	1406.3	1388.1–1424.6	<*0.001*	404.7	386.6–422.7	<*0.001*
G2	−9.7	−27.8–8.5	0.297	27.1	9.1–45.2	*0.003*
G3	−9.1	−27.0–8.8	0.320	11.1	−6.6–28.9	0.220
G4	−13.8	−42.6–15.1	0.350	4.1	−24.8–33.0	0.780
G5	−8.8	−30.7–13.1	0.430	11.6	−10.2–33.2	0.298
G6	−13.8	−31.5–3.9	0.126	2.4	−15.1–19.9	0.789
algorithm	−4.7	−6.5 to −2.9	<*0.001*	−0.80	−3.0–1.4	0.459
afternoon shift	71.0	46.3–95.7	<*0.001*	68.8	44.2–93.3	<*0.001*
irregular shift	25.4	9.0–41.8	*0.002*	31.1	14.6–47.6	<*0.001*
night shift	39.7	7.1–72.3	*0.017*	202.9	169.4–236.4	<*0.001*
retired	27.8	18.6–37.0	<*0.001*	43.5	34.3–52.8	<*0.001*
rotating shift	−4.2	−44.4–35.9	0.836	15.4	−18.27–57.70	0.446
split shift	46.3	11.2–81.4	*0.010*	52.3	16.6–87.9	*0.004*
age	−1.4	−1.9 to −1.0	<*0.001*	−0.5	−1.0 to −0.0	*0.030*
male	0.7	−6.8–8.1	0.859	−1.2	−8.6–6.3	0.758
Chinese American	7.9	−5.2–21.0	0.239	2.7	−10.4–15.8	0.683
African American	12.1	2.5–21.7	*0.013*	−0.1	−9.7–9.5	0.98
Hispanic	−6.1	−16.1–3.9	0.231	−11.6	−21.6 to −1.6	*0.023*

^a^Actigraphy group was analysed as a categorical variable with the group with best actigraphy quality, G7, as the reference. Algorithm was analysed as an indicator variable, with SOTs or WOTs from event buttons as reference. Job schedule was analysed as a categorical variable, with day job as the reference. Gender was modelled as an indicator variable, with female as the reference. Ethnicity was analysed as a categorical variable, with White as the reference.

## 4. Discussion

In this study, we improved a generic, unsupervised algorithm for detecting S/WOTs and validated it on a large actigraphy dataset. Originally developed for children’s actigraphy data collected by hip-worn ActiGraph sensors, this algorithm was seamlessly applied to adults’ actigraphy data collected by wrist-worn Actiwatch sensors. Validated against the self-reported S/WOT event markers, our proposed algorithm showed good consistency with the markers, resulting in a small bias of less than 4 min and an MAE less than 15 min in comparison with the markers. Using variance component models, we found that the between-subject variability and day-to-day variability within-subjects contributed by far the largest percentage to the total variances in S/WOTs. This highlights the necessity of SW cycle detection when analysing actigraphy data. While the between-subject variation can be partially modelled by certain covariates (e.g. age), individual habitual rhythms are largely random and cannot be learned well via population statistics. The large within-subject variation suggests that reporting average S/WOTs will lose considerable information about an individual’s sleep patterns.

So far, among studies that validate SW cycle algorithms, our study stands out in terms of sample size (*N* = 1857) and bias (underestimating SOTs and WOTs by 3.6 min and 0.8 min, respectively). Loock *et al.* [20] compared Munich Actimetry Sleep Detection Algorithm against event markers from data collected in 34 adolescents and 28 young adults, and their algorithm underestimated both SOT and WOT for 21 min. Werner *et al.* [34] compared actigraphy S/WOTs estimated by Actiwatch-4 against sleep diary collected in 50 kindergarten children, and found actigraphy underestimated SOTs by 5 min and WOTs by 6 min. Leister *et al.* [35] compared their SW detection algorithm against sleep logs, and found their algorithm (CREA) on average underestimated sleep time by 24 min and overestimated wake time by 27 min. Nauha *et al.* [36] compared S/WOTs from Oura ring and Polar Active against sleep diary in 108 adults, and found Oura ring overestimated bedtime and wake-up time by 3.6 min and 16.2 min, respectively, while Polar Active overestimated bedtime and wake-up time by 5.4 min and 18.9 min, respectively.

Our proposed algorithm has several advantages. Firstly, this top-down approach converts the SW cycle detection problem into a CP detection problem within bounded regions, which are the estimated circadian cycles. In contrast to previous methods that focused on supervised learning methods, the assumptions of CircaCP focus on the commonality of sleep patterns in humans. Following these assumptions, CircaCP captures the circadian cycles via a cosinor model and the day-to-day variability via CP detection bounded by each circadian cycle. Therefore, this method is directly transferable to other types of actigraphy, given apparent circadian rhythms and reasonably identified data distributions. For example, activity counts from Actiwatch and ActiGraph, although based on different proprietary algorithms, are continuous random variables with long-tail distributions, hence can both be modelled well by Gamma distributions where the dispersion (i.e. the inverse of the shape parameter) is explicitly estimated. For activity count data that are discrete and generated from a counting process (e.g. activity counts measured by infrared room occupancy sensors, or step counts from FitBit sensors), CP detection algorithms based on Poisson distributions may achieve better detection accuracy.

Secondly, the only parameter that needs to be chosen in advance is the threshold to dichotomize the cosinor curve. Although we used a set of priors to initialize the cosinor model, those values are related to the circadian rhythms of human beings, thus can be applied to other datasets without tuning. Since the proposed algorithm circumvents the laborious collection of sleep labels and training classifiers for actigraphy datasets collected by various devices with different configurations, this automated approach reduces the overall overhead in developing SW cycle detection algorithms.

Lastly, we elected to use the less computationally demanding cosinor model instead of the Hill-transformed cosinor model in our previous publication [24], so that the curve fitting step was more robust and much faster. Moreover, we improved the previous algorithm by a second round CP searching procedure, so that the misidentified CPs could be better located in the second round. Since all of these steps can be done in linear time (O(n)), with the algorithms implemented in Matlab 2021a using Intel i7 4-core CPU at 3.4 GHz with 16.0GB RAM, it takes less than 15 min to complete the SW cycle detection on 1930 subjects’ actigraphy data.

One limitation of using actigraphy for S/WOT detection is that the S/WOTs are effectively time-to-bed and time-to-get-up, whereas the precise time when the body physiologically and/or cognitively switches off is unattainable. For subjects who have afterhours sedentary behaviour, actigraphy-based SW detection can underestimate their SOTs by a large margin (e.g. 19.00 can be identified as the SOT if the subject stays in bed after dinner until falling asleep). Our finding that the bias in SOT detected by CircaCP is behind SOT reported by markers partially confirmed this underestimation. Still, the framework of CircaCP (i.e. identifying CPs within a circadian cycle) can also be applied to synchronous heart rate data for even more precise S/WOT estimation. Another limitation is that for certain subjects with severely irregular circadian rhythms (i.e. subjects whose actigraphy do not have SW cycles), the detection results contained large errors, as identified by the error detection method. Although this is a small percentage (less than 2%) of the cohort, their poor regularity may be of greater interest in studies investigating the impact of occupation on health. Similarly, our current algorithm does not detect polyphasic sleep patterns (e.g. naps). However, with the diurnal activity segmented by our algorithm, we can extend on our algorithm to detect a period of sleep within the day as naps. Another alternative to enable finer segmentation to include polyphasic or severely interrupted sleep patterns is to develop HMM with mixtures of Gamma distributions. Our future work will focus on fusing information from different sensor modalities and other types of estimation method in order to detect polyphasic sleep patterns. Lastly, as within-subject S/WOTs are influenced by the weekend effect, not considering the weekday versus weekend factor in the variance component analysis model may have increased the contribution from within-subject variance. Future circadian rhythm analysis and sleep analysis should take the weekday versus weekend factor into account.

The applications of our proposed algorithm are wide. In essence, it provides a unified framework for accurately detecting SW cycles and S/WOTs. In sleep research, multiple sleep-related metrics, such as sleep duration and S/WOT variability, can be derived based on accurate S/WOTs [37]. For actigraphy analysis in general, CircaCP can also serve as a pre-processing step by segmenting the long sequences of actigraphy into two regions, within which time series are relatively stationary, and thus time-series models can be validly applied and metrics related to physical activity profiles can be accurately extrapolated [38].

## 5. Conclusion

We proposed a generic algorithm for detecting SW cycles using unlabelled minute-by-minute actigraphy data. Validated against event markers in a large population cohort with actigraphy data collected from free-living environment, our algorithm shows an excellent agreement with event markers and that it can be readily applied to analyse free-living actigraphy data collected on various populations.

## Data Availability

The actigraphy dataset is publicly available and can be downloaded at National Sleep Research Resource’s website with registered access: https://sleepdata.org/datasets/mesa. The code is available on Zenodo [39].
